# Metacognitive Prompts Influence 7- to 9-Year-Olds’ Executive Function at the Levels of Task Performance and Neural Processing

**DOI:** 10.3390/bs15050644

**Published:** 2025-05-09

**Authors:** Colin Drexler, Philip David Zelazo

**Affiliations:** Institute of Child Development, University of Minnesota—Twin Cities, Minneapolis, MN 55455, USA

**Keywords:** metacognition, executive function, reflection, error-monitoring, post-error slowing, event-related potentials, ERN, N2

## Abstract

To elucidate the role of metacognitive reflection in the development of children’s executive function (EF) skills, the current study examined relations among implicit and explicit forms of metacognition in 7- to 9-year-olds during performance based on the Dimensional Change Card Sort (DCCS), while experimentally manipulating the propensity to reflect on the task. Results showed that instructions to reflect led to improved task accuracy and better metacognitive control, but only in younger children, likely because older children were already engaging in reflection. Individual differences in trait mindfulness were related to a similarly reflective mode of responding, characterized by improved task accuracy and metacognitive control. In contrast, articulatory suppression impaired children’s task accuracy and metacognitive monitoring. Additionally, simply asking children to make metacognitive judgments without extra instructions decreased the amplitude of event-related potential (ERP) indices of error detection (the error-related negativity; ERN) and conflict detection (the N2). Finally, individual differences in trait anxiety were related to larger Pe amplitudes. Taken together, the current findings reinforce theoretical frameworks integrating metacognition and EF and highlight the shared influence of metacognitive reflection across multiple levels of analysis.

## 1. Introduction

As children acquire the executive function (EF) skills involved in regulating their own cognition and behavior in the service of goals, they show an increased capacity to engage in deliberate reasoning and intentional action. EF skills are well-studied, and early individual differences in these skills predict numerous developmental outcomes, ranging from academic success (e.g., Cortés Pascual et al., 2019; Spiegel et al., 2021) to social and emotional wellbeing (e.g., Stucke & Doebel, 2024) to mental health and flourishing (e.g., Yang et al., 2022; Zelazo, 2020). In general, children can use cognitive skills or strategies with or without metacognitive awareness that they are doing so (Flavell, 1979), and a growing number of theoretical proposals address the role of metacognition in EF skills and their development (e.g., Chevalier et al., 2015; Lyons & Zelazo, 2011; Roebers, 2017). EF skills are often intentional and depend on some degree of reflective awareness (Zelazo, 2015). According to several hierarchical models of the development of intentional action and reasoning, metacognitive reflection on the use of cognitive skills, including EF skills, is important for noticing a problem and attempting to solve it intentionally using top-down processes that inevitably compete with more habitual or reflexive bottom-up influences on behavior. Reflection is arguably a matter of degree, however—there are levels of consciousness or reflective awareness (e.g., Demetriou et al., 2018; Marcovitch & Zelazo, 2009; Overton & Ricco, 2011; Zelazo, 2004). At one extreme, children may automatically change their behavior with little or no awareness that the modification occurred, and at the other, they may purposely select a course of action with full awareness of the interplay among their goals, behaviors, and thoughts. Between the extremes, however, are gradations of awareness that have been argued to depend on iterative degrees of information processing and the hierarchical embedding of interpretive schemata (Zelazo, 2015).

Traditionally, models of metacognition (e.g., Flavell, 1979; Nelson & Narens, 1994) have identified three metacognitive subcomponents: declarative knowledge, procedural monitoring, and procedural control. Declarative knowledge is defined as one’s explicit beliefs about cognition and the factors that influence cognition across a variety of contexts. On the other hand, explicit procedural metacognition, comprising both procedural monitoring and procedural control, corresponds to skills—things one has learned to do in particular contexts, and as such, it is always context-dependent to some degree. Whereas procedural monitoring refers to being aware of one’s current cognitive state, procedural control refers to the regulation of ongoing cognitive activities in light of one’s declarative knowledge and/or procedural monitoring. Procedural monitoring is typically studied by comparing a participant’s explicit subjective estimate of their own performance (whether predictively or retrospectively) against their actual performance and also by asking how confident the participant is about this estimate (e.g., Lyons & Ghetti, 2011; Schraw, 2009). Procedural control is usually measured as the extent to which these metacognitive monitoring judgments actually influence a participant’s subsequent behavior, for example by measuring whether participants selectively withdraw incorrect answers when given the opportunity to do so (e.g., Roebers & Spiess, 2017).

Research on the development of metacognition has historically focused on other aspects of cognition (e.g., memory, learning, or perception) besides EF skills and has remained relatively independent from research on the development of EF skills. More recently, studies have attempted to link metacognition to EF, but these studies have used distinct tasks to assess these two constructs, and they have yielded inconsistent results regarding the nature of metacognition–EF associations. Though some studies have found significant relations between EF and procedural metacognition (e.g., Kälin & Roebers, 2022), others have only found associations with declarative metacognition (e.g., Marulis & Nelson, 2021) or with certain components of EF (e.g., Bryce et al., 2015). Unlike EF, which is a relatively domain-general skill (Zelazo & Carlson, 2023), procedural metacognition is highly domain-specific, always corresponding to a specific context. Therefore, it is unclear whether to expect relations between these two constructs when comparing metacognition in one context, such as during a memory task (e.g., Geurten et al., 2016) or a spelling task (e.g., Roebers et al., 2012), to EF performance in the context of a distinct measure. Although domain-general EF skills have been revealed as developmental precursors to later domain-specific metacognition (Roebers et al., 2012), there is evidence suggesting that improved metacognitive skills in turn influence the way in which children choose to employ their still-developing EF skills, indicating a bidirectional relation between the two constructs (Roebers, 2017). Several studies, for example, have found that metacognitive training and support can improve children’s EF performance (e.g., Espinet et al., 2013; Hadley et al., 2020; Pozuelos et al., 2019).

Also, few studies on metacognition and EF have assessed all three subcomponents of metacognition simultaneously, but doing so is necessary to understand whether declarative knowledge, procedural monitoring, and procedural control differentially relate to EF skills. Finally, few studies have examined explicit metacognitive knowledge and confidence judgments in relation to more indirect indications of metacognition, such as post-error slowing (de Mooij et al., 2022), which is the tendency to respond more slowly on trials directly following an error, and which seems to imply some (potentially implicit) subjective awareness of that error, as well as an influence of that awareness on behavior (i.e., slowing down).

Neural responses such as event-related potentials (ERPs) represent other important and possibly implicit signatures of error monitoring and metacognition. For example, the error-related negativity (ERN) and error positivity (Pe) components of the ERP are reliable electrophysiological responses that occur within milliseconds of making an error on a cognitive task (Davies et al., 2004; Hajcak et al., 2005). The ERN appears first, usually within 100 ms following an error, while the Pe appears later, peaking at around 200 to 500 ms following an error. The ERN is believed to reflect the initial sense that an observed outcome is worse than expected (Holroyd & Coles, 2002), whereas the Pe might reflect a subsequent evaluation of this error. These neural correlates of error monitoring are proposed to represent the preliminary stage of hierarchical, self-reflective processing—the first iteration of interpreting one’s own actions in terms of one’s goals (Zelazo, 2015).

Finally, other ERP components such as the stimulus-locked N2 are also relevant to reflection and EF skills. The N2 response is larger when there is greater conflict, and it is also associated with less efficient deployment of EF skills (e.g., Lo, 2018). Larger N2 amplitudes are seen in children who fail to switch on the dimensional change card sort (DCCS; Zelazo, 2006), for example, which suggests that these children detect a conflict and keep on detecting that conflict but fail to resolve it (Espinet et al., 2012). The N2 amplitude decreases as children get older, but it is better predicted by performance based on independent measures of EF than by age *per se* (Lamm et al., 2006).

Individual differences in anxiety (e.g., Moser et al., 2013) and mindfulness (e.g., Kaunhoven & Dorjee, 2017) have also been found to relate to these indices of error monitoring, as well as to performance on EF tasks (Mak et al., 2018). It is believed that anxious children, who might be more likely to be vigilant about errors, display heightened conflict detection (N2) and error detection (ERN), as manifested by larger ERP amplitudes based on EF tasks (Moser et al., 2013). In contrast, children high in mindfulness or nonjudgmental present-moment awareness (Kabat-Zinn, 1994) might be less likely to worry about potential errors or ruminate on past errors and may display smaller N2 and ERN amplitudes as a result (Kaunhoven & Dorjee, 2017). 

A number of developmental relations have been observed between proactive metacognitive reflection—pausing and thinking twice before responding—and EF skills. For example, there is evidence that 4-year-old children can be taught to pause and reflect on what they are about to do and that reflection training improves their EF performance (e.g., Espinet et al., 2013). This finding suggests that proactive reflection can have a causal influence on EF skills. Moreover, starting around 5 to 7 years of age, children typically start making a speed–accuracy trade-off; they spontaneously slow down in order to maintain accuracy, which implies an increase in metacognitive reflection and proactive EF (Davidson et al., 2006). A longitudinal study of 5- to 7-year-olds (*N* = 425) found cross-lagged associations from slower reaction times (RTs) based on the DCCS at ages 5 and 6 years to one-year later improvements in accuracy (Dumont et al., 2022), consistent with the suggestion that children first reflect and slow down their RT in order to achieve accurate responding, and only then do they begin to improve efficiency and speed (through practice) while maintaining high accuracy. A similar developmental pattern has been observed in children learning to read (e.g., word recognition and reading comprehension), where children first reach an accuracy threshold and only then show increases in efficiency (e.g., Karageorgos et al., 2020).

During this developmental period, children also become more likely to spontaneously prepare proactively on a continuous performance task in which pairs of stimuli are presented sequentially and children can (but are not required to) prepare for the second stimulus in the pair after seeing the first. The likelihood of spontaneous proactive control increases between 5 and 10 years (e.g., Chevalier et al., 2015), and larger amplitude P3B waveforms (associated with working memory) have been recorded in trials involving proactive versus reactive control (Troller-Renfree et al., 2020). Whereas most children around 10 years old of age proactively engage EF skills (i.e., actively maintain goal-relevant information in mind before responding) in the context of cognitive tasks, children under 6 years old typically only do so when specifically instructed to prepare (Gonthier & Blaye, 2022), when incentivized by changing task demands (Chevalier et al., 2015), or when required to metacognitively monitor their own performance (Hadley et al., 2020). Another study showed that 7- to 8-year-olds rapidly improved their proactive reflection skills when they were given repeated practice with a task that required it (Heemskerk & Roebers, 2024). Taken together, these findings suggest that, in addition to EF capacity itself, metacognitive decisions regarding when to reflect and when to respond automatically are part of what differentiates the flexible, adaptive self-regulation typical of adolescents and adults from the inflexibility and perseveration characteristic of younger children. It may be the case that children who display poor EF skills are lacking metacognitive skills around using EF, more so than lacking EF skills *per se*. In this view, late childhood (e.g., 7 to 9 years) represents a period of transition between exercising EF skills reactively (e.g., following an error) and more spontaneous proactive reflection, allowing for a greater complexity of hierarchical representations and for proactive engagement of EF skills.

To investigate how (1) different subcomponents of explicit metacognition (knowledge, monitoring, and control), (2) an indirect indication of metacognition (post-error slowing), and (3) event-related potential (ERP) correlates of error monitoring and conflict detection (the ERN, Pe, and N2 ERP components) relate to EF skills measured behaviorally, the current study measured children’s EF performance and metacognition in the context of the same EF task, the DCCS (Zelazo et al., 2013), while experimentally manipulating children’s propensity to reflect prior to each trial. Children were randomly assigned to one of four conditions for a version of the DCCS modified to allow for periodic metacognitive confidence judgments: one condition was designed to encourage reflection, one (articulatory suppression) was designed to preclude reflection, and there were two control conditions. Articulatory suppression (i.e., the verbalization of task-irrelevant words) has been found to impair performance on EF tasks in children (Fatzer & Roebers, 2012), presumably by preventing linguistically mediated reflection and self-regulation (e.g., Emerson & Miyake, 2003; Luria, 1959). One control condition (MC Only) included only those instructions necessary to understand the task (DCCS and metacognitive questions) and allowed us to examine the effects of reflection-relevant instructions (reflection vs. articulatory suppression) on performance on the metacognitive questions. The second control condition was a no-metacognition (No MC) condition in which the periodic metacognitive judgments were replaced with simple factual questions (e.g., “*What color is the text in this sentence?*”). This allowed us to examine the extent to which metacognitive prompts themselves might elicit reflection and affect accuracy and RT on the DCCS. Procedural monitoring and control were assed using widely used procedures (e.g., Lyons & Ghetti, 2011; Roebers & Spiess, 2017; Schraw, 2009).

First, we hypothesized that making metacognitive judgments would improve EF performance and influence brain activity related to error monitoring (smaller amplitude ERN and larger amplitude Pe) and the efficiency of conflict detection (smaller amplitude N2). Second, we hypothesized that reflection instructions would improve EF and metacognitive performance and modulate these same neural markers of error monitoring and conflict detection in a similar way. Third, we hypothesized that suppression instructions would have the opposite effects, impairing EF and metacognitive performance and influencing the same neural markers in the opposite way (larger ERN, smaller Pe, and larger N2). Finally, given the evidence of positive relations between mindfulness and EF performance (Mak et al., 2018) and negative relations between anxiety and EF (Moran, 2016), we hypothesized that, regardless of the condition, anxiety and mindfulness would predict children’s task-directed metacognitive and EF performance, along with associated neural indicators of this performance, in a way consistent with the suggestion that mindfulness increases performance and anxiety attenuates it (e.g., Shields et al., 2016).

## 2. Materials and Methods

### 2.1. Participants

Participants included 119 7- to 9-year-old children (*M* = 8.53 years, *SD* = 0.86, range = 7.05–9.99, 69 male) and their caregivers recruited through the UMN Institute of Child Development participant pool. Eligibility criteria involved being a typically developing, literate English speaker within the appropriate age range. Participants whose parents reported an ADHD or autism diagnosis, for example, were excluded from participation. Participants were 2% African American, 83% Caucasian, 1% Asian, 12% more than one ethnicity, and 2% other ethnicity, as reported by the parents/guardians. Additionally, 5% of participants of any ethnicity were Hispanic/Latino. The median household income was $175,000–$199,999. Parental consent was obtained for all child participants. For compensation, all children received a t-shirt and a piece of candy, while parents received a $5 gift card.

### 2.2. Procedure

Children and their parent/guardian visited the University of Minnesota campus for a single study session lasting 1 h. Informed consent was obtained from the parent/guardian in a private room, and verbal assent was obtained from the child. Children were fitted with an electroencephalography (EEG) cap and then completed a metacognitive version of the DCCS (see below) in a separate testing room. Meanwhile, parents/guardians completed questionnaires in another room. After children completed the task, the EEG cap was removed, and they returned to the room with their parent/guardian to complete child questionnaires. One participant refused the EEG cap and completed behavioral measures only.

### 2.3. Measures

#### 2.3.1. Parent Questionnaires

Questionnaires were presented using REDCap on a tablet computer. The Developmental Social Cognitive Neuroscience Lab’s Family Information Questionnaire included 39 questions and asked parents/guardians about their family’s demographic information, such as the family income level, ages and genders of their child’s siblings, ethnicity, and parental education level.

The Screen for Child Anxiety Related Disorders-Parent version (SCARED-p; Birmaher et al., 1997) included 41 statements about their child’s anxiety, and parents indicated on a scale from 0 (not true) to 2 (very true) how true each statement was about their child. Example items include “My child is shy” and “My child worries about going to school”. Internal consistency for the SCARED-p was excellent (Cronbach’s α = 0.91) in the current sample.

#### 2.3.2. Child Questionnaires

The first child-report questionnaire was the Screen for Child Anxiety Related Disorders-Child version (SCARED-c; Birmaher et al., 1997). This is a self-report version of the SCARED-p validated for school-age children. This survey included the same 41 items as the parent version, although the statements involved first-person pronouns rather than references to “my child”. Example items include “I am shy” and “I worry about going to school”. Internal consistency for the SCARED-c was good (Cronbach’s α = 0.89) in the current sample. All analyses include a composite of SCARED-p and SCARED-c scores.

The second child-report questionnaire was the Mindful Attention Awareness Scale—Child version (MAAS-C; Lawlor et al., 2014). The MAAS-C is a self-report measure of trait mindfulness or the tendency to be aware of one’s thoughts and actions in the present moment that is validated for school-age children. It includes 15 questions that ask children to rate how frequently they engage in various forms of mindful attention, on a scale from 1 (almost never) to 6 (almost always). Children were also able to select “I don’t understand” as a response option on all items. Example items include “I rush through activities without being really attentive to them” and “I can’t stop thinking about the past or the future”. Internal consistency for the MAAS-C was acceptable (Cronbach’s α = 0.77) in the current sample.

#### 2.3.3. Child Interview

Immediately following completion of the DCCS, children were given an adapted version of the Metacognitive Knowledge Interview (McKI; Marulis et al., 2016). The McKI was originally designed to assess declarative metacognition in younger (3- to 5-year-old) children in the context of a problem-solving task. We modified the language to be developmentally appropriate for 7- to 9-year-olds and explicitly about children’s performance on the DCCS. Sample items include “If you tried to match the pictures as fast as possible, would it be easier? Why or why not?” To capture 7- to 9-year-olds’ more mature metacognitive and linguistic abilities, we also added a follow-up question of “How do you know that?” after each item, resulting in 12 total items. Data from each item were scored on a scale from 0–2, corresponding to responses that were not at all metacognitive (0), partially metacognitive (1), and fully metacognitive (2). For example, responses that referred to the child’s cognitive capacity, the difficulty of the task, or strategies used on the task (e.g., “It would be harder, because you need time to think, and if you do it that fast, you might not get it correct”) were coded as fully metacognitive. Responses that referred to cognitive capacity, task difficulty, or strategies in an incomplete way (e.g., “It would be harder, because you could get it wrong”) were coded as partially metacognitive. Finally, responses that did not refer to cognitive capacity, task difficulty, or strategies (e.g., “I don’t know”) were coded as not at all metacognitive (Marulis et al., 2016). One-quarter of participants’ interview responses were double-coded by independent examiners, and the intra-class coefficient was 0.79 (95% CI [0.58, 0.90]), indicating good reliability.

#### 2.3.4. Metacognitive DCCS

**Figure A1:**
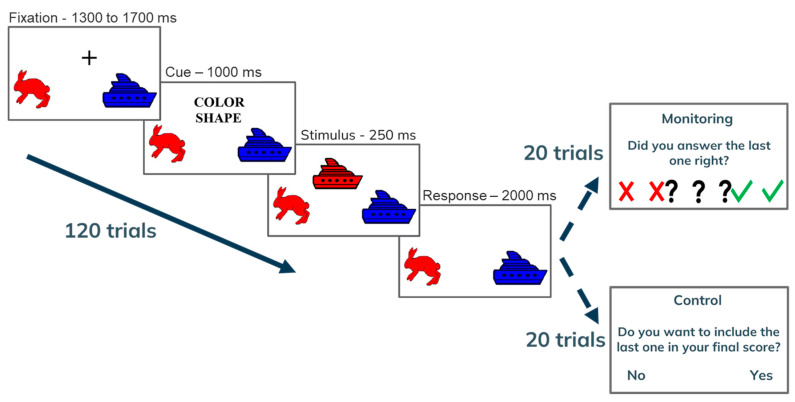
Schematic depicting the timeline of a trial for the metacognitive DCCS, with MC monitoring and MC control questions that appeared every 2–5 trials in a pseudo-random order.

A new metacognitive version of the DCCS was designed based on the mixed block of the NIH Toolbox version of the Dimensional Change Card Sort (DCCS; Zelazo et al., 2013). In this task, which was presented to children on a computer, children are instructed to sort virtual images (e.g., red rabbit, blue boat) according to one dimension (e.g., color) and then switch to sorting the same images according to another dimension (e.g., shape), with the sorting dimension varying unpredictably across trials. Each trial consists of an 800 ms inter-trial interval, a fixation cross displayed for a random interval between 1300 and 1700 ms, a cue (either the word “shape” or “color”) displayed for 1000 ms, a test stimulus displayed for 250 ms, and finally, once the stimulus disappeared, a 2000 ms response window (Appendix A Figure A1). After a pseudo-randomly varying number of trials (between 2 and 5 trials), participants had 10,000 ms to respond to one of two explicit metacognitive questions that were displayed until the participant responded (or until 10,000 ms elapsed). To assess procedural metacognitive monitoring, children were asked, “*Did you answer the last one right?*” with a 5-point pictorial scale from 0 (definitely wrong) to 1 (definitely right). Intermediate options included 0.25 (probably wrong), 0.5 (not sure), and 0.75 (probably right). To assess procedural metacognitive control, children were asked “*Do you want to include the last one in your final score?*”, with “Yes” and “No” as available options. The DCCS task consisted of 120 sorting trials, 40 of which included an attached metacognitive prompt, either monitoring (20 trials) or control (20 trials). Children were randomly assigned to one of four instruction conditions for the Metacognitive DCCS. The first condition was the *MC Only* condition, which consisted of the metacognitive DCCS (including periodic metacognitive questions) but with no special instructions, only those necessary to understand the task. The second condition, *MC + Reflection*, consisted of the metacognitive DCCS with extra instructions that children should “pause and think about which of the two games [they’re] playing” instead of “answering without thinking” (Espinet et al., 2013). The third condition, *MC + Suppression*, consisted of the metacognitive DCCS with extra instructions that children should talk to themselves while playing, repeating aloud the syllable “da-” in tune with an 80 bpm metronome (Fatzer & Roebers, 2012). The fourth condition was a *no-metacognition* (*No MC*) control condition in which the 40 metacognitive questions on the DCCS were replaced with simple factual questions (e.g., “*What color is the text in this sentence?*”). Finally, the same 80 bpm metronome sounded across all conditions, and trial lengths and counts remained identical across all conditions.

Children began the task with an instructional phase in which images were sorted by shape and color sequentially (five trials each); then, they received practice based on a mixed block in which the sorting dimension varied on each trial (six trials). Participants also received practice with the metacognitive prompts (six trials each), before finally beginning the test block of 120 trials. It was during this initial practice phase in which participants in the MC + Reflection and MC + Suppression conditions received their extra instructions. Participants in these conditions did not receive additional prompts during the test block.

#### 2.3.5. EEG Acquisition and Preprocessing

EEG was acquired using a 128-channel HydroCel Geodesic Sensor Net and Net Station 5.4.2 (Electrical Geodesic, Inc., Eugene, OR, USA). EEG analysis was performed using the EEGLAB toolbox (Delorme & Makeig, 2004) and ERPLAB (Lopez-Calderon & Luck, 2014). All EEG data were preprocessed in MATLAB R2024a using the Harvard Automated Processing Pipeline for Electroencephalography (HAPPE; Gabard-Durnam et al., 2018). During EEG acquisition, the 128 channels were sampled at 500 Hz, and electrode impedances were maintained below 50 kΩ. Within HAPPE, data were re-referenced to an average reference and band-pass filtered from 1 Hz to 30 Hz. Data were segmented into epochs from −250 ms to 800 ms relative to the stimulus presentation and response onset. Bad channels were removed before wavelet-enhanced independent component analysis (W-ICA) if the normed joint probability of the average log power fell more than 3 *SD* from the mean (Gabard-Durnam et al., 2018). These bad channels were later interpolated using spherical interpolation, and bad segments were interpolated using FASTER (Nolan et al., 2010). The average number of bad channels per participant was 9 out of 128, or 7%.

### 2.4. Data Analysis

#### 2.4.1. Data Preparation

To prepare the behavioral data from the Metacognitive DCCS, we first calculated participants’ overall accuracy. Then, we calculated the median reaction time (RT) of correct trials in milliseconds, excluding trials with RTs faster than 100 ms. DCCS RT was measured as the latency between the onset of the test stimulus and a participant’s key press response. To calculate DCCS post-error slowing, we subtracted participants’ DCCS RT based on each pre-error trial from their DCCS RT based on the associated post-error trial and calculated the mean duration of slowing across all errors (Dutilh et al., 2012). A higher (and positive) DCCS post-error slowing score (in ms) indicates the extent to which children responded more slowly on trials immediately following error trials than they did on trials immediately before error trials.

We then computed two probability scores to assess the accuracy of children’s procedural metacognitive monitoring and metacognitive control (Fleming & Lau, 2014; Harvey, 1997). For monitoring, this was calculated as one minus the squared difference between each participant’s rating of the probability that their responses on selected trials were accurate (from 0 [definitely wrong] to 1 [definitely right]) and each participant’s actual accuracy based on those trials. Thus, we first categorized trials according to a participant’s probability ratings (0, 0.25, 0.5, 0.75, 1) and then examined the mean accuracy within each category of the trial. Based on metacognitive monitoring trials in which participants judged that they were “probably right” (i.e., 0.75), for example, one would expect 75% of such items to be correct if participants displayed perfect monitoring. We did comparable analyses for metacognitive control. Among metacognitive control trials that participants chose *not* to include in their final score (i.e., 0), 0% should be correct. Greater discrepancies between mean correctness within a probability rating and that probability rating itself indicate deviations from metacognitive accuracy. For ease of interpretation, we reverse-coded these scores so that higher scores indicate greater accuracy of procedural metacognitive monitoring and control and smaller discrepancies between subjective and objective performance.

#### 2.4.2. ERP Analyses

To calculate the ERN and Pe amplitudes, response-locked epochs (−250 ms to 800 ms) were baseline corrected to the period −250 to −100 ms prior to the response, in order to avoid overlap with the motor response and beginning of the ERN signal. Participants who did not have at least 6 usable incorrect trials were not included in response-locked analyses (*n* = 11; Olvet & Hajcak, 2009). In our sample, the average number of correct trials included was 93, and the average number of incorrect trials was 27. We defined the ERN as the mean difference in amplitude between correct and incorrect trials from 25 to 100 ms post-response and the Pe as the mean difference in amplitude between correct and incorrect trials from 200 to 500 ms post-response. We measured the ERN at electrode clusters Fz (electrodes 11, 19, 18, 16, 10, and 4 based on our 128-channel net) and FCz (electrodes 6, 13, 12, 5, and 112) and found that the ERN response was maximal at Fz, so further analyses were conducted based on the Fz electrode cluster. The Pe was found to be maximal at the Pz electrode cluster (electrodes 62, 61, 67, 72, 77, and 78).

To calculate the N2, stimulus-locked epochs (−250 ms to 800 ms) were baseline corrected to the period −250 to 0 ms prior to the stimulus presentation. Unlike the response-locked ERPs, the stimulus-locked N2 included a longer baseline period, extending all the way to stimulus presentation. The N2 was calculated as the mean amplitude 275 to 350 ms post-stimulus presentation, and because it was not found to differ in our sample between switch and repeat trials, all trial types were averaged together and included both correct and incorrect responses (e.g., Espinet et al., 2012, 2013). The N2 was found to be maximal at electrode cluster FCz.

**Figure A2:**
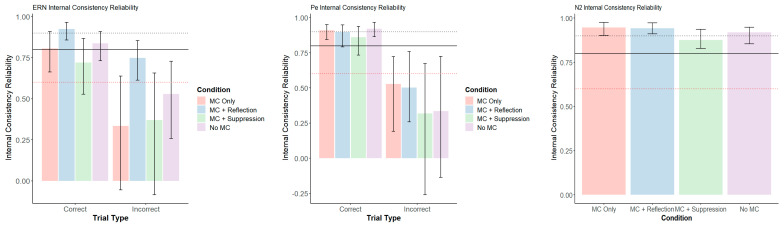
Overall internal consistency reliability across conditions for the ERN, Pe, and N2 components’ mean amplitudes. The error bars represent 95% confidence intervals from the resampling distribution. The red dotted line represents the threshold for acceptable data quality (0.60), the black solid line represents the threshold for good data quality (0.80), and the black dotted line represents the threshold for excellent data quality (0.90).

**Figure A3:**
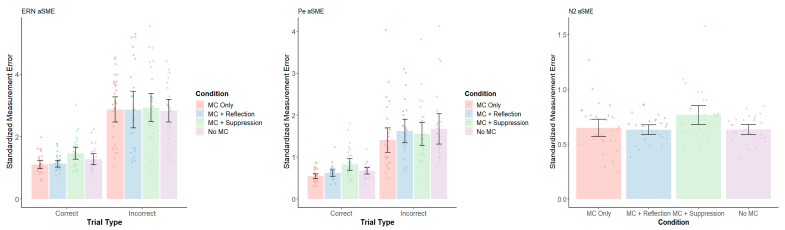
Averaged standardized measurement error (*aSME*) output across conditions for the ERN, Pe, and N2 components. The error bars represent 95% confidence intervals of the variability between individuals. Dots represent the individual participants. *aSME* = Analytic Standard Measurement Error.

For each of our three ERP components (ERN, Pe, N2), we calculated the internal consistency reliability (Appendix A Figure A2) and analytic standard measurement error (aSME; Appendix A Figure A3) as metrics of data quality across conditions using the READIE toolbox (Xu et al., 2024).

#### 2.4.3. Analytic Plan

First, we conducted descriptive analyses and standardized all study variables. Then, to test whether the act of making explicit metacognitive judgments influenced EF performance, implicit metacognition, and potential neural correlates of EF and metacognition, we fitted a series of ordinary least squares linear regression models in which participants’ DCCS accuracy, DCCS RT, DCCS post-error slowing, and the amplitudes of the ERN, Pe, and N2 components were independently regressed on the condition (MC Only vs. No MC) and a series of covariates including the family demographic information, age, mindfulness, anxiety, and declarative metacognition.

Second, to test whether reflection instructions influenced EF performance, implicit and explicit metacognition, and potential neural correlates of EF and metacognition, we fitted a series of ordinary least-squares regression models in which participants’ EF performance and ERPs (as described above), as well as metacognitive monitoring and control scores, were independently regressed based on the condition (MC + Reflection condition vs. MC Only condition vs. MC + Suppression condition) and the same series of covariates. In order to maintain the assumption of linearity for ordinary least-squares regression, metacognitive control scores were square-root transformed, which kept their 0 to 1 scale the same. All other models met the assumptions of linearity, homoscedasticity, and normality without the need for transformations.

Finally, as an exploratory analysis, we extracted residuals from these multiple regression equations to determine correlations among the various outcome variables at the levels of task performance, metacognitive ratings, and scalp-electrical neural activity while accounting for the shared effects of demographic variables, declarative metacognition, and individual differences in mindfulness and anxiety. Because these analyses were exploratory, we employed a Benjamini–Hochberg correction for multiple comparisons for these correlations (Benjamini & Hochberg, 1995).

## 3. Results

### 3.1. Task-Level Results

**Figure 1:**
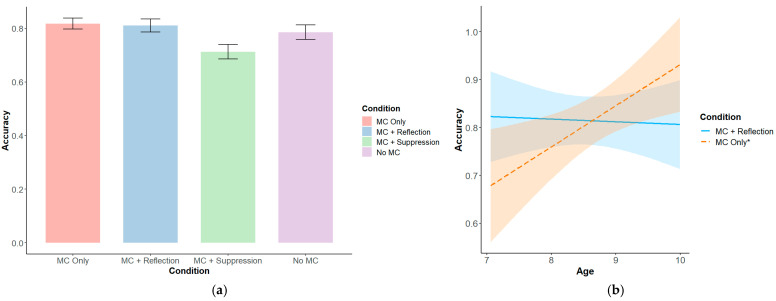
(**a**) DCCS accuracy by experimental condition; (**b**) simple slopes of the interaction between reflection prompts and age based on DCCS accuracy. An asterisk (*) indicates that the slope is significantly different from zero (*p* < 0.05).

Participants’ mean DCCS accuracy was 0.78 (*SD* = 0.14), their mean DCCS RT was 708 ms (*SD* = 219 ms), and their mean DCCS post-error slowing was 51 ms (*SD* = 242 ms). Relative to the MC Only condition, participants in the MC + Suppression condition displayed significantly worse DCCS accuracy (*β* = −0.70, *SE* = 0.26, *p* = 0.008; see Figure 1a). There was also a significant interaction between the effect of the MC + Reflection condition (vs. the MC Only condition) and age on DCCS accuracy, such that younger children received a larger benefit from reflection instructions relative to older children (*β* = −0.65, *SE* = 0.30, *p* = 0.03) (Figure 1b). Regardless of the condition, older children (*β* = 0.61, *SE* = 0.23, *p* = 0.01) and children with higher self-reported mindfulness scores (*β* = 0.23, *SE* = 0.09, *p* = 0.01) showed significantly higher DCCS accuracy. No other covariates were significantly related to DCCS accuracy (see Table A1).

There were no significant effects of condition or any covariates on DCCS RT or DCCS post-error slowing (all *p*s > 0.05; see Table A1).

### 3.2. Metacognitive-Level Results

Participants’ mean procedural metacognitive monitoring score was 0.89 (*SD* = 0.18), and their mean procedural metacognitive control score was 0.81 (*SD* = 0.19). The mean declarative metacognitive knowledge score was 12.81 (*SD* = 3.68).

**Figure 2:**
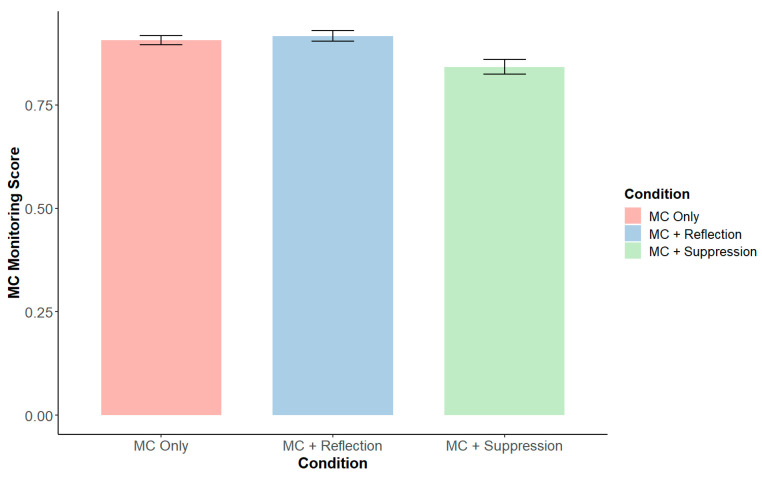
Metacognitive monitoring by experimental condition.

**Figure 3:**
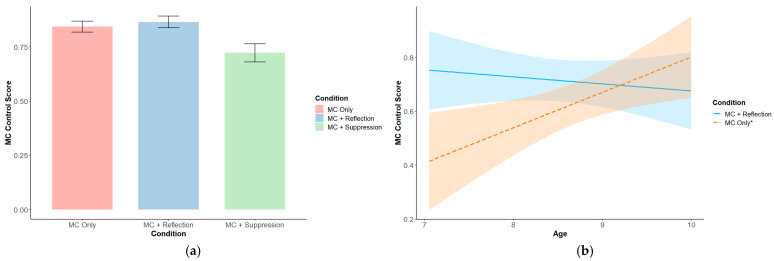
(**a**) Metacognitive control score by experimental condition; (**b**) simple slopes of the interaction between reflection prompts and age based on the metacognitive control score. An asterisk (*) indicates that the slope is significantly different from zero (*p* < 0.05).

Relative to the MC Only condition, participants in the MC + Suppression condition displayed a lower metacognitive monitoring score (*β* = −0.76, *SE* = 0.28, *p* = 0.009) (Figure 2), indicating larger discrepancies between their DCCS accuracy and their judgments of DCCS accuracy. For procedural metacognitive control, there was a significant negative interaction between the effect of reflection instructions (vs. the MC Only condition) and age, such that there was a smaller effect of reflection instructions in older children (*β* = −0.72, *SE* = 0.30, *p* = 0.02) (Figure 3b). Regardless of the condition, children demonstrated higher metacognitive control scores with age (*β* = 0.60, *SE* = 0.23, *p* = 0.01), indicating an increase in the likelihood of withholding incorrect responses and including correct ones. Also, regardless of the condition, children with higher mindfulness scores (*β* = 0.22, *SE* = 0.10, *p* = 0.04) and higher declarative metacognitive knowledge (*β* = 0.32, *SE* = 0.10, *p* = 0.003) had higher metacognitive control scores (see Table A1).

### 3.3. Neural-Level Results

**Figure 4:**
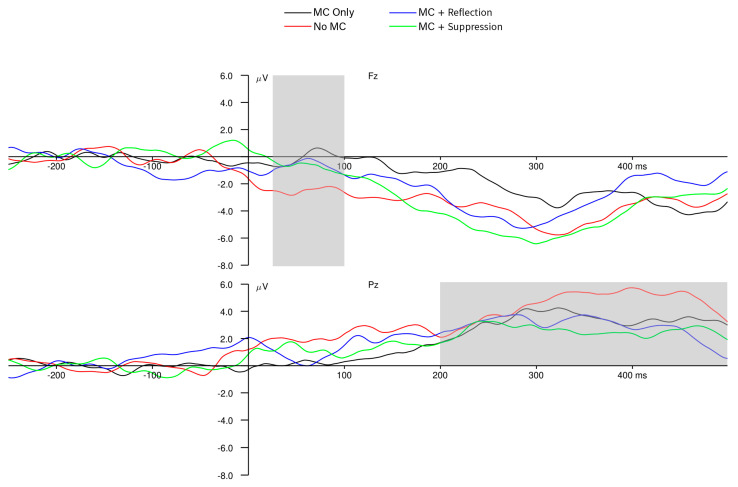
Response-locked ERPs (ERN and Pe components) by experimental condition. Shaded areas indicate the post-response temporal regions during which the ERN (top panel) and the Pe (bottom panel) were measured. Participants in the MC Only condition displayed a significantly smaller (i.e., less negative) ERN amplitude than those in the No MC condition (*β* = 0.67, *SE* = 0.31, *p* = 0.03), reflecting the attenuating influence of metacognitive questions on the ERN amplitude.

**Figure 5:**
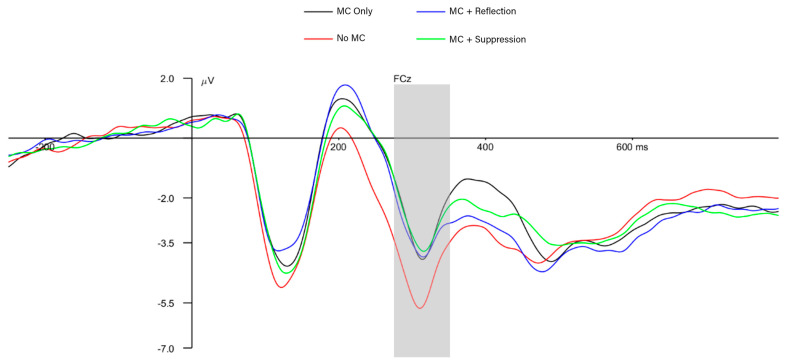
Stimulus-locked ERPs (the N2 component) by experimental condition. Shaded areas indicate the post-response temporal regions during which the N2 was measured. Participants in the MC Only condition demonstrated significantly smaller (i.e., less negative) N2 amplitudes than children in the No MC condition (*β* = 0.93, *SE* = 0.29, *p* = 0.002), reflecting the attenuating influence of metacognitive questions on the N2 amplitude.

As indicated in Figure 4 and Figure 5, the mean amplitude of participants’ ERN was −0.87 μV, the mean Pe amplitude was +3.50 μV, and the mean N2 amplitude was −3.35 μV. Participants in the MC Only condition displayed a significantly smaller (i.e., less negative) ERN amplitude than those in the No MC condition (*β* = 0.67, *SE* = 0.31, *p* = 0.03; Figure 4), consistent with our hypothesis that making metacognitive judgements would attenuate ERN amplitudes. Also, as predicted, children with greater anxiety (regardless of the experimental condition), displayed a significantly larger Pe amplitude (*β* = 0.40, *SE* = 0.11, *p* < 0.001), and children in the MC Only condition (*β* = 0.93, *SE* = 0.29, *p* = 0.002) demonstrated significantly smaller (i.e., less negative) N2 amplitudes than children in the No MC condition (Figure 5). Finally, children with higher parent-reported income demonstrated significantly larger N2 amplitudes than children with lower parent-reported income (*β* = −0.07, *SE* = 0.03, *p* = 0.02; see Table A1).

### 3.4. Residual Correlations Among Multi-Level Dependent Variables

Among the eight primary DCCS-derived dependent variables, three were measured at the task level (DCCS accuracy, DCCS RT, DCCS post-error slowing), two at the metacognitive level (procedural monitoring and procedural control), and three at the neural level (ERN, Pe, N2). Among these, participants’ DCCS accuracy was significantly positively related to their DCCS RT (ρ = 0.25, *p* = 0.048), such that higher DCCS accuracy was associated with slower RTs. Additionally, DCCS accuracy was positively related to metacognitive monitoring (ρ = 0.61, *p* < 0.001) and metacognitive control (ρ = 0.41, *p* = 0.001), such that higher DCCS accuracy was associated with better procedural metacognitive monitoring and control. Participants’ metacognitive control was positively related to their metacognitive monitoring (ρ = 0.42, *p* = 0.001) and positively related to the ERN amplitude (ρ = 0.33, *p* = 0.027), such that those with better metacognitive control displayed smaller (less negative) ERN amplitudes (Table 1).

## 4. Discussion

Overall, in line with our hypotheses, our experimental conditions influenced performance across three levels of analysis: task performance, metacognitive ratings, and scalp-electrical neural activity. First, as predicted, the reflection instructions elicited a mode of responding that was more accurate and associated with greater metacognitive control (more accurately withdrawing incorrect responses), but only in younger children. Relative to the MC Only condition (no special instructions but including metacognitive questions), both DCCS accuracy and metacognitive control in the MC + Reflection condition displayed a significant negative interaction with age, indicating that the facilitative effect of reflection instructions on DCCS accuracy and metacognitive control was greater for younger children than for older. By age 9 years, many children in the MC Only condition were likely beginning to reflect spontaneously, prepare proactively, and respond more accurately even without explicit instruction. But among 7-year-olds, explicit instruction to reflect evidently induced metacognitive proactive reflection, resulting in more accurate responding. Interestingly, children in any experimental condition and of any age who were high in self-reported mindfulness also displayed a similar reflective style of performance. They too responded more accurately and showed better metacognitive control, supporting the validity of the mindfulness measure in children in this age range. Additionally, higher DCCS accuracy was related to slower DCCS RTs, aligning with models of EF development according to which children must first slow down their RT in order to achieve accurate responding but then begin to improve efficiency and speed while maintaining high accuracy (Dumont et al., 2022), a developmental phenomenon also seen in children learning to read (Juul et al., 2014; Karageorgos et al., 2020).

Second, as predicted, suppression instructions had the opposite overall effect as reflection instructions. Suppression was related to lower DCCS accuracy and worse procedural metacognitive monitoring scores. Impairments in children’s task-level performance due to a secondary distractor task are well studied (e.g., Fatzer & Roebers, 2012), but our findings demonstrate that children’s metacognitive monitoring of their own task performance is also diminished when they are distracted and prevented from using language in the service of metacognition.

Partially in line with our hypotheses, participants’ procedural metacognitive control scores improved with age, but their procedural metacognitive monitoring scores did not. Additionally, declarative metacognitive knowledge, derived from their McKI scores, was only positively related to procedural metacognitive control, but not procedural metacognitive monitoring or any task-level measures of EF performance. Children may only become able to show effective procedural metacognitive control after they begin to metacognitively monitor their own knowledge in the first place. In contrast to procedural metacognitive control (which was strongly correlated with age), even the youngest 7-year-olds in our sample demonstrated good procedural metacognitive monitoring (and monitoring was not correlated with age), so a ceiling effect may have precluded any correlation between monitoring and declarative metacognitive knowledge.

Contrary to our hypotheses, we did not find any associations between the experimental condition or any covariates and DCCS RT or with our RT-based indicator of implicit metacognition, DCCS post-error slowing. These null findings may be due to the nature of the task, which involved periodic stoppages to ask metacognition questions. Post-error slowing may only be manifested when there is an uninterrupted stream of responses, not when there are already built-in periodic “slow-down” periods.

Results at the neural level displayed a complementary, but not overlapping, pattern to those at the task and metacognitive levels of analysis. Contrary to our hypotheses, there was no effect of reflection or suppression instructions on the amplitudes of any of our measured ERP components, but there *was* a difference between the No MC condition and the MC Only condition for ERN and N2 amplitudes. Simply asking children to make metacognitive judgments was associated with smaller ERN and N2 amplitudes, consistent with the suggestion that these metacognitive questions (included in the MC Only, MC + Reflection, and MC + Suppression conditions) themselves elicit proactive preparation for future trials. In contrast, the act of making metacognitive judgments (i.e., MC Only vs. No MC condition) did not influence any task- or metacognitive-level outcomes, but as previously outlined, there were numerous behavioral effects of reflection and suppression instructions. Children in the No MC condition, who were *not* prompted to assess their own task performance periodically throughout the task, demonstrated larger ERN and N2 amplitudes, reflecting a larger and less efficient brain response to errors and to stimulus-related conflict, respectively. This may indicate that the act of making metacognitive judgments prepared the brain in advance for errors. There was no need for such a large reactive alerting response (i.e., the ERN) or response to the detection of conflict (i.e., the N2) when children were already proactively thinking metacognitively and prepared to resolve the conflict. Because the ERN occurs so soon after errors are initiated, the amplitude of this waveform likely represents a relatively reactive evaluative response to the errors (Overbeek et al., 2005). The fact that there was no difference between the later Pe scores across conditions adds to this interpretation; the ERN and Pe likely reflect distinct aspects of error processing, the initial conflict evaluation and further conscious reflection on the error, respectively (Overbeek et al., 2005). The proactive metacognitive evaluation may have only attenuated the initial reactive error detection (ERN) and initial conflict detection but not subsequent processing of that conflict. Regardless of the condition, children who displayed better procedural metacognitive control also demonstrated a smaller ERN response, further suggesting that proactive preparation based on metacognitive control decisions improves the efficiency of reactive error monitoring and reduces the need for a large ERN response to error detection. Finally, we found that a larger Pe amplitude, but not ERN amplitude, was related to individual differences in anxiety. In previous work, ERN–anxiety relations have been found more consistently than Pe–anxiety relations (Boen et al., 2022), so this novel finding warrants further consideration in order to disentangle the nuances of how anxious children’s error preoccupation manifests in the brain.

Our study design has numerous strengths. Most notably, to the best of our knowledge, this was the first study to measure children’s metacognition in the context of an EF task, which proved, despite the frequent stoppages, to be sufficiently challenging for our participants, yielding sufficient variability in performance to test our hypotheses. Despite growing recognition of the theoretical relations between EF and metacognition, existing empirical studies have utilized different tasks to measure the constructs (Marulis et al., 2020). Because the metacognition of memory, perception, or learning might follow distinct developmental trajectories from metacognition of EF, and because EF and metacognitive skills are likely, to some extent, context-dependent (e.g., Zelazo & Carlson, 2023), it is crucial to examine how these skills interact with each other within the same task. Additionally, our study captures three subcomponents of metacognition (declarative knowledge, procedural monitoring, and procedural control) and reveals among them both unique developmental trends and unique associations with EF skills. In addition to task-level and metacognitive-level performance, this study also measured neural functioning using ERPs. Understanding how conflict and error monitoring function at multiple levels of analysis is critical for more comprehensive models of self-regulation and metacognition.

Several limitations of the present study should be noted, however. First, the convenience sample was relatively homogeneous, primarily comprised of children from a large urban area whose parents are relatively educated, affluent, and white. It will be crucial to determine whether our results are also found in samples of children outside of this specific convenience sample. For example, the age at which children spontaneously begin to reflect proactively might vary across a more representative sample of the population. Additionally, higher parent-reported income predicted a larger N2 amplitude in our sample, which is intriguing given the general trend that N2 amplitudes decrease as children develop (Lo, 2018). This result may be related to the lack of income variability in our sample and underscores the fact that our findings should be interpreted cautiously until they are replicated in a more diverse sample.

Additionally, the task used in this study, the DCCS, primarily challenges children’s cognitive flexibility and inhibitory control (i.e., the working memory demands are minimized because, in each trial, children are instructed to sort by a particular dimension). Although we were able successfully to demonstrate the effectiveness of measuring EF and metacognition within the same task, it is still unclear whether the observed findings would translate to tasks that place larger demands on working memory. That said, different aspects of EF are largely overlapping (i.e., undifferentiated) in children in this age range (e.g., Laureys et al., 2022). Also, our measures of procedural metacognitive monitoring and control, although commonly used in studies with structured cognitive tasks (e.g., Lyons & Ghetti, 2011; Roebers & Spiess, 2017; Schraw, 2009), primarily assess the accuracy of metacognitive judgments, not the intention to engage one’s metacognitive capabilities. Our future work will aim to measure the relations between metacognition and EF in a more unstructured, naturalistic context in which children have more freedom to exercise (or not) metacognitive monitoring and control.

Another possible limitation is that the DCCS and especially the Metacognitive DCCS is more complex than the measures used in the majority of studies examining neural markers of error monitoring, which tend to use simpler inhibitory control tasks where participants are required to follow a single pair of rules (e.g., press in response to a letter, but do not press when it is an X). Our findings regarding the ERN, Pe, and N2 might be specific to the task used, and this warrants further study. Finally, to reduce burnout in our child participants, our task length (120 trials) was somewhat shorter than many studies investigating error monitoring in older children and adults. Because ERP analyses require many trials for adequate power, it will be important to replicate the current findings using a task with more trials (potentially yielding more errors). Appendix A Figure A2 and Figure A3 reveal that incorrect trials in our task had markedly worse internal consistency reliability and Analytic Standard Measurement Error (*aSME*) than correct trials, data quality metrics that may be improved with a longer task.

## 5. Conclusions

The present study examined the relations among EF performance, metacognitive awareness, and neural indicators of error monitoring and EF skills within the same task, while experimentally manipulating the propensity to reflect, by facilitating reflection and interfering with it. Brief reflection instructions were related to improved performance at the task-performance and metacognitive-judgment levels of analysis, articulatory suppression was related to worse performance at the task and metacognitive levels, and the simple act of making metacognitive judgments influenced neural markers of error monitoring and conflict processing. Taken together, the current findings offer support for theoretical proposals integrating metacognition and EF (e.g., Lyons & Zelazo, 2011; Roebers, 2017; Zelazo, 2015) and highlight the influence of reflection on EF performance across multiple levels of analysis. Future work should extend these findings and investigate how longer-term training interventions designed to improve children’s EF skills could benefit from a more targeted focus on metacognitive skills like reflection (e.g., Espinet et al., 2013; Hadley et al., 2020; Pozuelos et al., 2019). When children use their EF skills reflectively and proactively and with increased metacognitive awareness, they are self-regulating in a more flexible and adaptive manner. And when children do not exercise their EF skills with metacognitive awareness, they do not harness their full potential for adaptive self-regulation.

## Figures and Tables

**Table 1:** Descriptive statistics and correlations among DCCS-derived dependent variables.

	*N*	Mean	*SD*	1.	2.	3.	4.	5.	6.	7.	8.
1. DCCS accuracy	119	0.78	0.14	-							
2. DCCS RT	119	708	219	0.25 *	-						
3. DCCS PES	116	51	242	−0.07	0.06	-					
4. MC monitoring	90	0.89	0.18	0.61 ***	0.14	−0.06	-				
5. MC control	90	0.81	0.19	0.41 **	0.11	−0.13	0.42 ***	-			
6. ERN amplitude	107	−0.87	5.04	−0.07	−0.02	0.05	0.03	0.33 *	-		
7. Pe amplitude	107	3.50	4.44	0.21	0.21	−0.22	0.07	0.13	−0.16	-	
8. N2 amplitude	118	−3.35	3.22	0.01	0.18	0.03	−0.09	0.15	0.11	0.07	-

* = *p* < 0.05, ** = *p* < 0.01, *** = *p* < 0.001. *N* = the number of participants with complete data per measure. RT = reaction time. PES = post-error slowing. MC = metacognitive.

## Data Availability

The original data presented in the study are openly available in the Data Repository for the University of Minnesota (DRUM) at https://hdl.handle.net/11299/269942 (accessed on 30 April 2025).

## Abbreviations

The following abbreviations are used in this manuscript:

aSME	Analytic Standard Measurement Error
DCCS	Dimensional Change Card Sort
EEG	Electroencephalography
EF	Executive Function
ERN	Error-related Negativity
ERP	Event-related Potential
HAPPE	Harvard Automated Processing Pipeline for Electroencephalography
MAAS	Mindful Attention Awareness Scale
MC	Metacognitive
McKI	Metacognitive Knowledge Interview
Pe	Error Positivity
RT	Reaction Time
SCARED	Screen for Child Anxiety Related Disorders

## Appendix A

**Table A1:** Standardized estimates from a series of ordinary least-squares regression equations.

	*N*	No-MC	No-MC × Age	Reflection	Reflection × Age	Suppression	Suppression × Age	Age	Anxiety	Mindfulness	MC Knowledge	Gender	Income	Ethnicity
DCCS Accuracy	107	−0.18	0.03	0.07	−0.64 *	−0.70 **	−0.43	0.61 *	−0.05	0.23 *	0.11	−0.20	−0.01	−0.46
DCCS RT	107	0.09	0.05	−0.04	−0.28	0.24	−0.23	0.03	0.03	0.06	0.04	−0.39	0.02	−0.06
DCCS PES	104	0.30	−0.00	0.28	−0.16	−0.00	0.39	0.11	−0.02	0.09	0.11	−0.08	−0.03	0.10
MC Monitoring	82	-	-	0.25	−0.13	−0.76 **	−0.35	0.13	−0.08	0.01	0.18	0.06	0.02	0.22
MC Control	82	-	-	0.47	−0.72 *	−0.35	−0.25	0.60 *	0.15	0.22 *	0.32 **	−0.39	−0.02	0.10
ERN amplitude	96	−0.67 *	−0.42	−0.20	−0.11	−0.13	−0.20	0.04	0.12	0.06	−0.04	0.16	−0.05	0.03
Pe amplitude	96	0.56	0.10	−0.12	0.07	0.08	−0.03	0.15	0.40 ***	0.00	0.02	0.01	0.03	−0.29
N2 amplitude	106	−0.93 **	0.30	−0.55	−0.03	−0.40	0.05	−0.29	−0.02	0.01	−0.15	0.14	−0.07 *	0.07

* = *p* < 0.05, ** = *p* < 0.01, *** = *p* < 0.001. *N* = the number of participants included in each model, based on missing covariate and outcome measures. RT = reaction time. PES = post-error slowing. MC = metacognitive. No-MC = no metacognition condition. Reflection = reflection instruction condition. Suppression = articulatory suppression condition. All estimates for condition differences are relative to the MC Only condition, including the two-way interactions (e.g., No-MC × Age). Monitoring and Control scores are reverse-coded (i.e., smaller scores equal better metacognition), and the N2 and ERN are negative-going components (i.e., smaller scores equal a larger negative amplitude). Thus, negative estimates for these outcomes indicate that a given predictor was associated with better metacognition or larger ERP amplitudes, and vice versa.

## References

[B1-behavsci-15-00644] Benjamini Y., Hochberg Y. (1995). Controlling the false discovery rate: A practical and powerful approach to multiple testing. Journal of the Royal Statistical Society Series B: Statistical Methodology.

[B2-behavsci-15-00644] Birmaher B., Khetarpal S., Brent D., Cully M., Balach L., Kaufman J., Neer S. M. (1997). The screen for child anxiety related emotional disorders (SCARED): Scale construction and psychometric characteristics. Journal of the American Academy of Child & Adolescent Psychiatry.

[B3-behavsci-15-00644] Boen R., Quintana D. S., Ladouceur C. D., Tamnes C. K. (2022). Age-related differences in the error-related negativity and error positivity in children and adolescents are moderated by sample and methodological characteristics: A meta-analysis. Psychophysiology.

[B4-behavsci-15-00644] Bryce D., Whitebread D., Szűcs D. (2015). The relationships among executive functions, metacognitive skills and educational achievement in 5 and 7 year-old children. Metacognition and Learning.

[B5-behavsci-15-00644] Chevalier N., Martis S. B., Curran T., Munakata Y. (2015). Metacognitive processes in executive control development: The case of reactive and proactive control. Journal of Cognitive Neuroscience.

[B6-behavsci-15-00644] Cortés Pascual A., Moyano Muñoz N., Quílez Robres A. (2019). The relationship between executive functions and academic performance in primary education: Review and meta-analysis. Frontiers in Psychology.

[B7-behavsci-15-00644] Davidson M. C., Amso D., Anderson L. C., Diamond A. (2006). Development of cognitive control and executive functions from 4 to 13 years: Evidence from manipulations of memory, inhibition, and task switching. Neuropsychologia.

[B8-behavsci-15-00644] Davies P. L., Segalowitz S. J., Gavin W. J. (2004). Development of response-monitoring ERPs in 7-to 25-year-olds. Developmental Neuropsychology.

[B9-behavsci-15-00644] Delorme A., Makeig S. (2004). EEGLAB: An open source toolbox for analysis of single-trial EEG dynamics including independent component analysis. Journal of Neuroscience Methods.

[B10-behavsci-15-00644] Demetriou A., Makris N., Kazi S., Spanoudis G., Shayer M. (2018). The developmental trinity of mind: Cognizance, executive control, and reasoning. Wiley Interdisciplinary Reviews: Cognitive Science.

[B11-behavsci-15-00644] de Mooij S. M., Dumontheil I., Kirkham N. Z., Raijmakers M. E., van der Maas H. L. (2022). Post-error slowing: Large scale study in an online learning environment for practising mathematics and language. Developmental Science.

[B12-behavsci-15-00644] Dumont É., Castellanos-Ryan N., Parent S., Jacques S., Séguin J. R., Zelazo P. D. (2022). Transactional longitudinal relations between accuracy and reaction time on a measure of cognitive flexibility at 5, 6, and 7 years of age. Developmental Science.

[B13-behavsci-15-00644] Dutilh G., Vandekerckhove J., Forstmann B. U., Keuleers E., Brysbaert M., Wagenmakers E. J. (2012). Testing theories of post-error slowing. Attention, Perception, & Psychophysics.

[B14-behavsci-15-00644] Emerson M. J., Miyake A. (2003). The role of inner speech in task switching: A dual-task investigation. Journal of Memory and Language.

[B15-behavsci-15-00644] Espinet S. D., Anderson J. E., Zelazo P. D. (2012). N2 amplitude as a neural marker of executive function in young children: An ERP study of children who switch versus perseverate on the Dimensional Change Card Sort. Developmental Cognitive Neuroscience.

[B16-behavsci-15-00644] Espinet S. D., Anderson J. E., Zelazo P. D. (2013). Reflection training improves executive function in preschool-age children: Behavioral and neural effects. Developmental Cognitive Neuroscience.

[B17-behavsci-15-00644] Fatzer S. T., Roebers C. M. (2012). Language and executive functions: The effect of articulatory suppression on executive functioning in children. Journal of Cognition and Development.

[B18-behavsci-15-00644] Flavell J. H. (1979). Metacognition and cognitive monitoring: A new area of cognitive–developmental inquiry. American Psychologist.

[B19-behavsci-15-00644] Fleming S. M., Lau H. C. (2014). How to measure metacognition. Frontiers in Human Neuroscience.

[B20-behavsci-15-00644] Gabard-Durnam L. J., Mendez Leal A. S., Wilkinson C. L., Levin A. R. (2018). The Harvard Automated Processing Pipeline for Electroencephalography (HAPPE): Standardized processing software for developmental and high-artifact data. Frontiers in Neuroscience.

[B21-behavsci-15-00644] Geurten M., Catale C., Meulemans T. (2016). Involvement of executive functions in children’s metamemory. Applied Cognitive Psychology.

[B22-behavsci-15-00644] Gonthier C., Blaye A. (2022). Preschoolers can be instructed to use proactive control. Cognitive Development.

[B23-behavsci-15-00644] Hadley L. V., Acluche F., Chevalier N. (2020). Encouraging performance monitoring promotes proactive control in children. Developmental Science.

[B24-behavsci-15-00644] Hajcak G., Moser J. S., Yeung N., Simons R. F. (2005). On the ERN and the significance of errors. Psychophysiology.

[B25-behavsci-15-00644] Harvey N. (1997). Confidence in judgment. Trends in Cognitive Sciences.

[B26-behavsci-15-00644] Heemskerk C. H. H. M., Roebers C. M. (2024). Speed and accuracy training affects young children’s cognitive control. Journal of Cognition and Development.

[B27-behavsci-15-00644] Holroyd C. B., Coles M. G. (2002). The neural basis of human error processing: Reinforcement learning, dopamine, and the error-related negativity. Psychological Review.

[B28-behavsci-15-00644] Juul H., Poulsen M., Elbro C. (2014). Separating speed from accuracy in beginning reading development. Journal of Educational Psychology.

[B29-behavsci-15-00644] Kabat-Zinn J. (1994). Wherever you go, there you are: Mindfulness meditation in everyday life.

[B30-behavsci-15-00644] Karageorgos P., Richter T., Haffmans M. B., Schindler J., Naumann J. (2020). The role of word-recognition accuracy in the development of word-recognition speed and reading comprehension in primary school: A longitudinal examination. Cognitive Development.

[B31-behavsci-15-00644] Kaunhoven R. J., Dorjee D. (2017). How does mindfulness modulate self-regulation in pre-adolescent children? An integrative neurocognitive review. Neuroscience & Biobehavioral Reviews.

[B32-behavsci-15-00644] Kälin S., Roebers C. M. (2022). Longitudinal associations between executive functions and metacognitive monitoring in 5-to 8-year-olds. Metacognition and Learning.

[B33-behavsci-15-00644] Lamm C., Zelazo P. D., Lewis M. D. (2006). Neural correlates of cognitive control in childhood and adolescence: Disentangling the contributions of age and executive function. Neuropsychologia.

[B34-behavsci-15-00644] Laureys F., De Waelle S., Barendse M. T., Lenoir M., Deconinck F. J. (2022). The factor structure of executive function in childhood and adolescence. Intelligence.

[B35-behavsci-15-00644] Lawlor M. S., Schonert-Reichl K. A., Gadermann A. M., Zumbo B. D. (2014). A validation study of the mindful attention awareness scale adapted for children. Mindfulness.

[B36-behavsci-15-00644] Lo S. L. (2018). A meta-analytic review of the event-related potentials (ERN and N2) in childhood and adolescence: Providing a developmental perspective on the conflict monitoring theory. Developmental Review.

[B37-behavsci-15-00644] Lopez-Calderon J., Luck S. J. (2014). ERPLAB: An open-source toolbox for the analysis of event-related potentials. Frontiers in Human Neuroscience.

[B38-behavsci-15-00644] Luria A. R. (1959). The directive function of speech in development and dissolution. Word.

[B39-behavsci-15-00644] Lyons K. E., Ghetti S. (2011). The development of uncertainty monitoring in early childhood. Child Development.

[B40-behavsci-15-00644] Lyons K. E., Zelazo P. D. (2011). Monitoring, metacognition, and executive function: Elucidating the role of self-reflection in the development of self-regulation. Advances in Child Development and Behavior.

[B41-behavsci-15-00644] Mak C., Whittingham K., Cunnington R., Boyd R. N. (2018). Efficacy of mindfulness-based interventions for attention and executive function in children and adolescents—A systematic review. Mindfulness.

[B42-behavsci-15-00644] Marcovitch S., Zelazo P. D. (2009). A hierarchical competing systems model of the emergence and early development of executive function. Developmental Science.

[B43-behavsci-15-00644] Marulis L. M., Nelson L. J. (2021). Metacognitive processes and associations to executive function and motivation during a problem-solving task in 3–5 year olds. Metacognition and Learning.

[B44-behavsci-15-00644] Marulis L. M., Baker S. T., Whitebread D. (2020). Integrating metacognition and executive function to enhance young children’s perception of and agency in their learning. Early Childhood Research Quarterly.

[B45-behavsci-15-00644] Marulis L. M., Palincsar A. S., Berhenke A. L., Whitebread D. (2016). Assessing metacognitive knowledge in 3–5 year olds: The development of a metacognitive knowledge interview (McKI). Metacognition and Learning.

[B46-behavsci-15-00644] Moran T. P. (2016). Anxiety and working memory capacity: A meta-analysis and narrative review. Psychological Bulletin.

[B47-behavsci-15-00644] Moser J. S., Moran T. P., Schroder H. S., Donnellan M. B., Yeung N. (2013). On the relationship between anxiety and error monitoring: A meta-analysis and conceptual framework. Frontiers in Human Neuroscience.

[B48-behavsci-15-00644] Nelson T. O., Narens L., Metcalfe J., Shimamura A. P. (1994). Why investigate metacognition?. Metacognition: Knowing about knowing.

[B49-behavsci-15-00644] Nolan H., Whelan R., Reilly R. B. (2010). FASTER: Fully automated statistical thresholding for EEG artifact rejection. Journal of Neuroscience Methods.

[B50-behavsci-15-00644] Olvet D. M., Hajcak G. (2009). Reliability of error-related brain activity. Brain Research.

[B51-behavsci-15-00644] Overbeek T. J., Nieuwenhuis S., Ridderinkhof K. R. (2005). Dissociable components of error processing: On the functional significance of the Pe vis-à-vis the ERN/Ne. Journal of Psychophysiology.

[B52-behavsci-15-00644] Overton W. F., Ricco R. B. (2011). Dual–systems and the development of reasoning: Competence–procedural systems. WIREs Cognitive Science.

[B53-behavsci-15-00644] Pozuelos J. P., Combita L. M., Abundis A., Paz-Alonso P. M., Conejero Á., Guerra S., Rueda M. R. (2019). Metacognitive scaffolding boosts cognitive and neural benefits following executive attention training in children. Developmental Science.

[B54-behavsci-15-00644] Roebers C. M. (2017). Executive function and metacognition: Towards a unifying framework of cognitive self-regulation. Developmental Review.

[B55-behavsci-15-00644] Roebers C. M., Cimeli P., Röthlisberger M., Neuenschwander R. (2012). Executive functioning, metacognition, and self-perceived competence in elementary school children: An explorative study on their interrelations and their role for school achievement. Metacognition and Learning.

[B56-behavsci-15-00644] Roebers C. M., Spiess M. (2017). The development of metacognitive monitoring and control in second graders: A short-term longitudinal study. Journal of Cognition and Development.

[B57-behavsci-15-00644] Schraw G. (2009). A conceptual analysis of five measures of metacognitive monitoring. Metacognition and Learning.

[B58-behavsci-15-00644] Shields G. S., Sazma M. A., Yonelinas A. P. (2016). The effects of acute stress on core executive functions: A meta-analysis and comparison with cortisol. Neuroscience & Biobehavioral Reviews.

[B59-behavsci-15-00644] Spiegel J. A., Goodrich J. M., Morris B. M., Osborne C. M., Lonigan C. J. (2021). Relations between executive functions and academic outcomes in elementary school children: A meta-analysis. Psychological Bulletin.

[B60-behavsci-15-00644] Stucke N. J., Doebel S. (2024). Early childhood executive function predicts concurrent and later social and behavioral outcomes: A review and meta-analysis. Psychological Bulletin.

[B61-behavsci-15-00644] Troller-Renfree S. V., Buzzell G. A., Fox N. A. (2020). Changes in working memory influence the transition from reactive to proactive cognitive control during childhood. Developmental Science.

[B62-behavsci-15-00644] Xu W., Monachino A. D., McCormick S. A., Margolis E. T., Sobrino A., Bosco C., Franke C. J., Davel L., Zieff M. R., Donald K. A., Gabard-Durnam L. J., Morales S. (2024). Advancing the reporting of pediatric EEG data: Tools for estimating reliability, effect size, and data quality metrics. Developmental Cognitive Neuroscience.

[B63-behavsci-15-00644] Yang Y., Shields G. S., Zhang Y., Wu H., Chen H., Romer A. L. (2022). Child executive function and future externalizing and internalizing problems: A meta-analysis of prospective longitudinal studies. Clinical Psychology Review.

[B64-behavsci-15-00644] Zelazo P. D. (2004). The development of conscious control in childhood. Trends in Cognitive Sciences.

[B65-behavsci-15-00644] Zelazo P. D. (2006). The dimensional change card sort (DCCS): A method of assessing executive function in children. Nature Protocols.

[B66-behavsci-15-00644] Zelazo P. D. (2015). Executive function: Reflection, iterative reprocessing, complexity, and the developing brain. Developmental Review.

[B67-behavsci-15-00644] Zelazo P. D. (2020). Executive function and psychopathology: A neurodevelopmental perspective. Annual Review of Clinical Psychology.

[B68-behavsci-15-00644] Zelazo P. D., Anderson J. E., Richler J., Wallner-Allen K., Beaumont J. L., Weintraub S. (2013). II. NIH toolbox cognition battery (CB): Measuring executive function and attention. Monographs of the Society for Research in Child Development.

[B69-behavsci-15-00644] Zelazo P. D., Carlson S. M. (2023). Reconciling the context-dependency and domain-generality of executive function skills from a developmental systems perspective. Journal of Cognition and Development.

